# TCam-2 Cells Deficient for SOX2 and FOXA2 Are Blocked in Differentiation and Maintain a Seminoma-Like Cell Fate In Vivo

**DOI:** 10.3390/cancers11050728

**Published:** 2019-05-25

**Authors:** Daniel Nettersheim, Saskia Vadder, Sina Jostes, Alena Heimsoeth, Hubert Schorle

**Affiliations:** 1 Department of Urology, Urological Research Lab, Translational Urooncology, University Hospital Düsseldorf, 40225 Düsseldorf, Germany; Daniel.Nettersheim@med.uni-duesseldorf.de; 2 Department of Developmental Pathology, Institute of Pathology, Bonn University Medical School, 53127 Bonn, Germany; Saskia.Vadder@gmx.de (S.V.); Sina.Jostes@ukbonn.de (S.J.); Alena.Heimsoeth@uk-koeln.de (A.H.)

**Keywords:** germ cell tumors, seminoma, embryonal carcinoma, reprogramming, microenvironment, SOX2, FOXA2

## Abstract

Testicular germ cell tumors (GCTs) are very common in young men and can be stratified into seminomas and non-seminomas. While seminomas share a similar gene expression and epigenetic profile with primordial germ cells, the stem cell population of the non-seminomas, the embryonal carcinoma (EC), resembles malignant embryonic stem cells. Thus, ECs are able to differentiate into cells of all three germ layers (teratomas) and even extra-embryonic-tissue-like cells (yolk-sac tumor, choriocarcinoma). In the last years, we demonstrated that the cellular microenvironment considerably influences the plasticity of seminomas (TCam-2 cells). Upon a microenvironment-triggered inhibition of the BMP signaling pathway in vivo (murine flank or brain), seminomatous TCam-2 cells reprogram to an EC-like cell fate. We identified SOX2 as a key factor activated upon BMP inhibition mediating the reprogramming process by regulating pluripotency, reprogramming and epigenetic factors. Indeed, CRISPR/Cas9 SOX2-deleted TCam-2 cells were able to maintain a seminoma-cell fate in vivo for about six weeks, but after six weeks in vivo still small sub-populations initiated differentiation. Closer analyses of these differentiated clusters suggested that the pioneer factor FOXA2 might be the driving force behind this induction of differentiation, since many FOXA2 interacting genes and differentiation factors like *AFP*, *EOMES*, *CDX1*, *ALB*, *HAND1*, *DKK*, *DLK1*, *MSX1* and *PITX2* were upregulated. In this study, we generated TCam-2 cells double-deficient for *SOX2* and *FOXA2* using the CRISPR/Cas9 technique and xenografted those cells into the flank of nude mice. Upon loss of SOX2 and FOXA2, TCam-2 maintained a seminoma cell fate for at least twelve weeks, demonstrating that both factors are key players in the reprogramming to an EC-like cell fate. Therefore, our study adds an important piece to the puzzle of GCT development and plasticity, providing interesting insights in what can be expected in a patient, when GCT cells are confronted with different microenvironments.

## 1. Introduction

All type II testicular germ cell tumors (GCTs) derive from a common precursor lesion—the germ cell neoplasia in situ (GCNIS), which itself is thought to be the results of a defective primordial germ cell (PGC) development [1,2]. GCTs can be stratified into seminomas and non-seminomas [1]. Seminomas and embryonal carcinomas (ECs; the stem cell population of the non-seminomas) differ considerably in the histology, their gene expression profiles, and epigenetics. While ECs resemble malignant embryonic stem cells, seminomas are more similar to PGCs and GCNIS [1].

In the past years, we and others have shown that the cell line TCam-2 serves as a reliable proxy for seminomas and GCNIS. TCam-2 cells express typical primordial germ cell and GCNIS marker genes (*SOX17*, *PRAME*, *cKIT*, *TFAP2C*, *PRDM1*/*BLIMP1*) and show a typical GCNIS/seminoma morphology (big roundish cells with a big nucleus and clear cytoplasm) [3,4,5,6]. Additionally, we have shown that TCam-2 cells reprogram into an EC-like cell fate upon growth in a somatic microenvironment, like the murine flank or brain [4,7]. Molecular analyses revealed that reprogramming is initiated by inhibition of BMP signaling, subsequently leading to SOX2 induction that establishes the NODAL signaling cascade and upregulates several pluripotency, EC and reprogramming factors, like *GDF3*, *DNMT3B*, *JARID2*, *PRDM14*, *DPPA4* [7]. As a consequence of reprogramming, global DNA methylation levels strongly increase to levels comparable to EC cells, while DNA methylation levels specifically in EC-associated genes decrease considerably [7,8]. This suggests that TCam-2 cells show a remarkable plasticity and reprogramming can be considered complete since the transcriptome and the methylome are highly identical to EC. Using CRISPR/Cas9 gene editing we demonstrated that deletion of SOX2 severely impairs reprogramming [9]. Most TCam-2 cells lacking SOX2 maintain a seminoma-like morphology, gene expression and DNA methylation profile for at least six weeks [9]. Upon closer inspection, we detected in the xenografted area small nests of cells, which display downregulation of seminoma and pluripotency markers (*SOX17*, *TFAP2C*, *OCT3/4*) and upregulation of differentiation markers (*AFP*, *EOMES*, *FOXA2*, *HAND1*, *ALB*, *CDX1*, *APOA1/A2/B/C1/E/H/M*, *FGA/B/H/L1*, *HPX*, *FLRT3*, etc.) [9,10,11,12]. We noticed that many of the differentiation markers found upregulated interact with FOXA2. FOXA2 is a pioneer factor and regulator of cellular differentiation [9,13,14,15]. Thus, we hypothesized that FOXA2 might play an important role in the observed induction of differentiation during the in vivo growth of SOX2-deficient TCam-2, prompting us in this study to analyze the role of FOXA2 in this process.

## 2. Results

To test our hypothesis that FOXA2 is a crucial factor for induction of differentiation in *SOX2*-deficient TCam-2 cells in vivo, we generated TCam-2 cells deficient for *SOX2* and *FOXA2* using the CRISPR/Cas9 gene editing technique. To establish double-deficient cells, we utilized *SOX2*-deficient TCam-2 cells generated in a previous study [9] and re-transfected cells with the CRISPR/Cas9 components including three guideRNAs targeting the coding region of *FOXA2* (Figure S1A). *FOXA2* is encoded on chromosome 20, which has six copies in TCam-2 cells [5]. A successful deletion of all *FOXA2* alleles was verified by using a three primer pair PCR strategy (Figure S1B). Primer pair 1 (red arrows) flanking guideRNA 1, primer pair 2 (orange arrows) flanking guideRNA 3 and primer pair 3 (blue arrows) flanking all three guideRNAs (Figure S1B). Primer pair 1 amplifies a product of 240 bp in case of wildtype *FOXA2* or deletion of the region between guideRNA2 and guideRNA3. Primer pair 2 will amplify a product of 190bp in case of wildtype *FOXA2* or deletion of the region between guideRNA1 and guideRNA2. Primer pair 3 only results in an amplification of a 200 bp fragment upon deletion of the entire region spanning from guideRNA1 to guideRNA3. We generated three *FOXA2*-deficient TCam-2 cell clones (Figure S1B). Clones 1 to 3 harbor a homozygous deletion for *FOXA2* (clone 1: loss of the entire region between guideRNA1–3 on all alleles; clone 2: loss of the entire region between guideRNA1–3 on at least one allele, loss of the region between guideRNA1 and 2 on all alleles; clone 3: loss of the entire region between guideRNA1–3 on at least one allele, loss of the region between guideRNA2 and 3 on all alleles).

Next, we confirmed that *SOX2*- and *FOXA2*-deficient TCam-2 cells do not differ from the parental TCam-2 cells with regard to proliferation and gene expression of typical GCNIS/seminoma and differentiation markers. Within eleven days, proliferation rates did not differ between parental TCam-2, TCam-2 deficient for *SOX2* (TCam-2-Δ*SOX2*) and TCam-2 deficient for both, *SOX2* and *FOXA2* (TCam-2-Δ*SOX2*/*FOXA2*) (Figure S2). Additionally, strong and comparable expression of the seminoma and pluripotency markers *OCT3/4*, *NANOG*, *LIN28*, *SOX17*, *PRAME*, *PRDM1*/*BLIMP1* and *TFAP2C* was found in TCam-2, TCam-2-Δ*SOX2* and TCam-2-Δ*SOX2*/*FOXA2* cells (Figure S3A). As controls, 2102EP EC cells and PC3 prostate cancer cells (positive control for FOXA2 [16]) were included. In line with our hypothesis that FOXA2 is only upregulated during in vivo growth of TCam-2 cells driving their differentiation, FOXA2 expression was not detectable in all GCT cell lines in vitro. We confirmed absence of FOXA2 and SOX2 as well as expression of SOX17 and PRAME in TCam-2, TCam-2-Δ*SOX2* and TCam-2-Δ*SOX2*/*FOXA2* cells on protein level by western blotting (Figure S3B). Again, 2102EP and PC3 cells served as (positive and negative) controls. These findings demonstrate that expression of typical seminoma and pluripotency factors is not considerably affected by *SOX2*- and *FOXA2*-deficiency in TCam-2 cells and thus, deletion of *SOX2* and *FOXA2* does not affect the seminoma cell fate in vitro.

**Figure 1:**
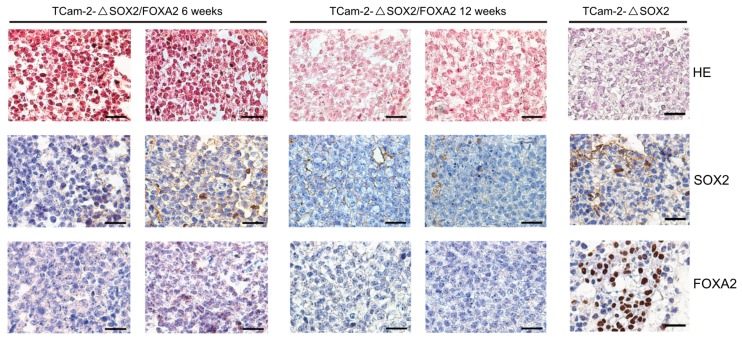
HE and IHC staining of SOX2 and FOXA2 in TCam-2-Δ*SOX2*/*FOXA2* tumor tissues six and twelve weeks after xenografting. TCam-2-ΔSOX2 tumor tissue served as control (six weeks in vivo). Scale bars: 200 μm.

**Figure 2:**
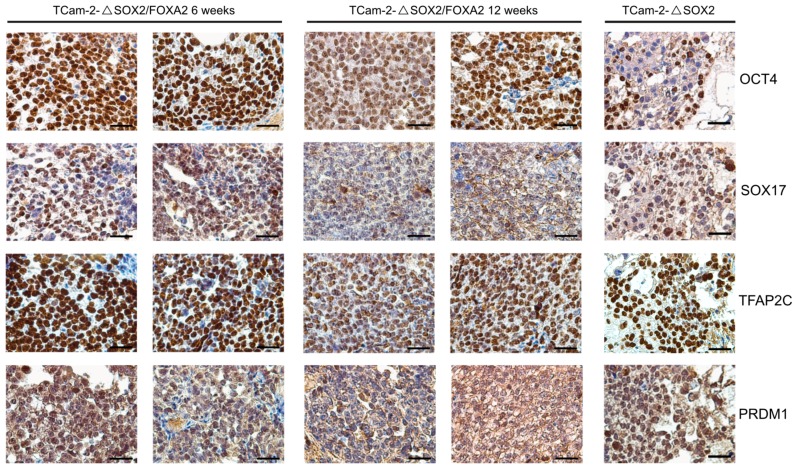
IHC staining of the pluripotency and seminoma markers OCT4, SOX17, TFAP2C and PRDM1 in TCam-2-Δ*SOX2*/*FOXA2* tumor tissues six and twelve weeks after xenografting. TCam-2-Δ*SOX2* tumor tissue served as control (six weeks in vivo). Scale bars: 200 μm.

**Figure 3:**
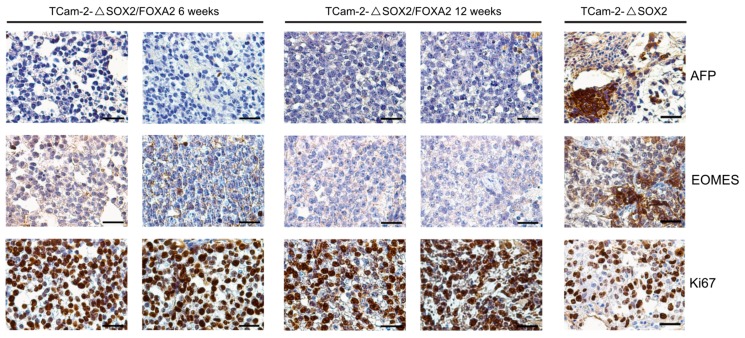
IHC staining of the differentiation markers AFP and EOMES and the proliferation marker Ki67 in TCam-2-Δ*SOX2*/*FOXA2* tumor tissues six and twelve weeks after xenografting. TCam-2-Δ*SOX2* tumor tissue served as control (six weeks in vivo). Scale bars: 200 μm.

To analyze the effect of a SOX2-/FOXA2-deficiency on TCam-2 cells in vivo, we xenografted TCam-2-Δ*SOX2* and TCam-2-Δ*SOX2*/*FOXA2* cells into the flank of nude mice and analyzed the tumor tissues after six and twelve weeks. By HE and immunohistochemical stainings (IHC), we observed that the tissue xenografts presented as homogenous tumor masses with a typical seminoma-like morphology, i.e., big roundish cells with a big nucleus, a clear cytoplasm and clearly distinguishable cellular boundaries (Figure 1). Additionally, we confirmed that TCam-2-Δ*SOX2*/*FOXA2* derived tumors were indeed negative for SOX2 and FOXA2 (Figure 1). Furthermore, TCam-2-Δ*SOX2*/*FOXA2* derived tumors stained positive for pluripotency and seminoma markers OCT3/4, SOX17, TFAP2C, and PRDM1/BLIMP1 (Figure 2), but were negative for the differentiation-markers AFP and EOMES (Figure 3). High numbers of Ki67-stained cells indicated that the tumor cells were highly proliferating (Figure 3). In contrast, TCam-2-Δ*SOX2* derived tumors were positive for FOXA2, AFP and EOMES (Figure 1 and Figure 3). Of note, an overview of IHC data of all five tumor tissues analyzed after twelve weeks is given in Figure S4.

**Figure 4:**
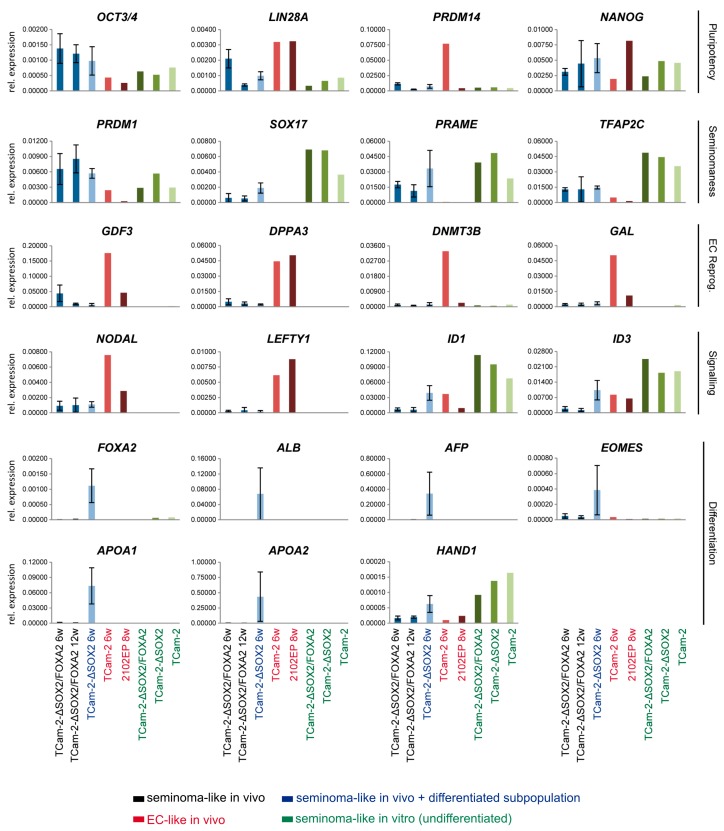
qRT-PCR analysis of indicated marker genes in TCam-2-Δ*SOX2*/*FOXA2* (six (*n* = 4) and twelve weeks (*n* = 5)) and TCam-2-Δ*SOX2* (six weeks (*n* = 4)). In vivo reprogrammed TCam-2 (TCam-2 6w) and in vivo grown 2102EP (2102EP 8w) as well as in vitro cultivated TCam-2-Δ*SOX2*/*FOXA2*, TCam-2-Δ*SOX2* and parental TCam-2 cells served as controls. Expression levels were normalized against *GAPDH*.

To confirm and extend these findings, we performed qRT-PCR analyses on the TCam-2-Δ*SOX2* and TCam-2-Δ*SOX2*/*FOXA2* tumors (Figure 4). As controls, xenografted 2102EP and TCam-2 cells reprogrammed to an EC were included. Additionally, in vitro cultivated TCam-2-Δ*SOX2*, TCam-2-Δ*SOX2*/*FOXA2* and parental TCam-2 were analyzed as controls. We found expression of pluripotency factors *OCT3/4*, *LIN28* and *NANOG* in the TCam-2-Δ*SOX2*/*FOXA2* tumor tissues, but not of PRDM14, which is strongly upregulated in TCam-2 cells, which were reprogrammed to an EC-like cell fate (Figure 4). Expression of typical seminoma markers (*PRDM1*/*BLIMP1*, *SOX17*, *PRAME*, *TFAP2C*) was also detectable in TCam-2-Δ*SOX2*/*FOXA2* tumor tissues, in contrast to EC reprogramming factors (*GDF3*, *DPPA3*, *DNMT3B*, *GAL*), which could only be detected in 2102EP in vivo or reprogrammed TCam-2 cells (Figure 4). In a previous study, we demonstrated that the reprogramming of TCam-2 cells into an EC-like fate is triggered by inhibition of BMP signaling leading to SOX2 induction [7]. SOX2 enables establishment of the NODAL signaling cascade by binding to the essential NODAL co-factors LEFTY1 and CRIPTO, but not NODAL itself [9].

Inhibition of BMP signaling is also detectable in TCam-2-Δ*SOX2* cells in vivo, since induction of SOX2 is downstream of BMP inhibition [9]. In TCam-2-Δ*SOX2* cells in vivo, expression of its co-factors *LEFTY1* and *CRIPTO* is not induced, while *NODAL* is slightly upregulated [9]. In line with these results, in our qRT-PCR analysis TCam-2-Δ*SOX2* and TCam-2-Δ*SOX2*/*FOXA2* tumors show downregulation of the BMP signaling effectors *ID1* and *ID3* and only slight upregulation of *NODAL*, but not of its essential co-factor *LEFTY1* (Figure 4). Finally, in TCam-2-Δ*SOX2*/*FOXA2* cells in vivo expression of *FOXA2* and several FOXA2-associated differentiation factors (*ALB*, *AFP*, *EOMES*, *APOA1*, *APOA2*, *HAND1*) is not induced (Figure 4). Expression of these factors is strongly upregulated in xenografted TCam-2-Δ*SOX2* cells (Figure 4). These findings demonstrate that TCam-2-Δ*SOX2*/*FOXA2* cells maintain their seminoma-like cell fate for at least twelve weeks and do neither initiate reprogramming nor differentiation. This clearly shows that while SOX2 is necessary to induce reprogramming, FOXA2 (in TCam-2-Δ*SOX2*) is central to trigger differentiation.

## 3. Discussion

**Figure 5:**
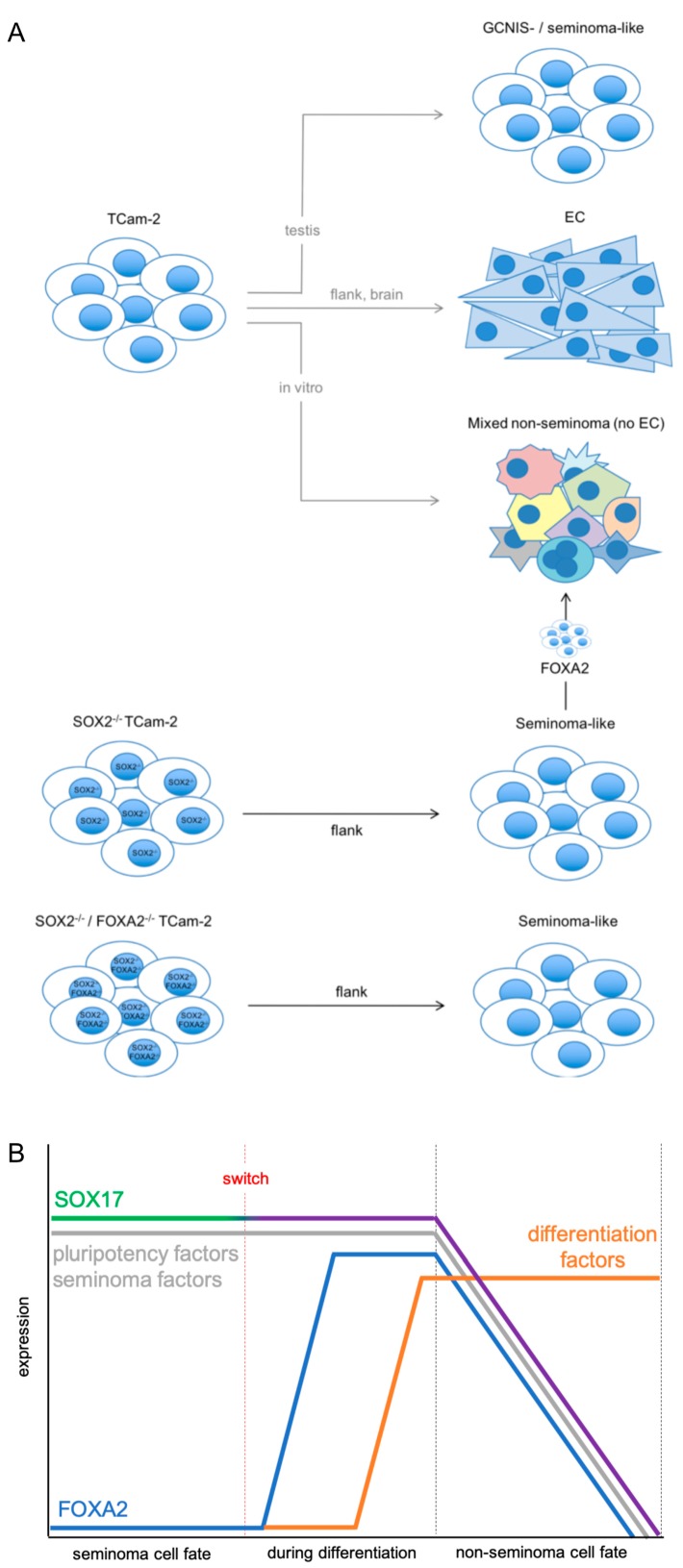
(**A**) Model summarizing the influence of the microenvironment on the cell fate of TCam-2 cells and the role of SOX2 and FOXA2 in these processes (based on [9]). (**B**) Illustration of the molecular effects associated with the switch of SOX17 from a pluripotency to a differentiation-inducing factor during in vivo growth of TCam-2-Δ*SOX2*.

In this study, we analyzed the role of FOXA2, a pioneer factor and inducer of differentiation, in the microenvironment-triggered reprogramming of TCam-2 cells into an EC. TCam-2 cells grow as a seminoma either in vitro or after transplantation into the testis of nude mice (Figure 5A) [3,4]. In contrast, TCam-2 reprogram to an EC in vivo upon contact with a somatic microenvironment, e.g., the flank or brain (Figure 5A) [4,7]. Initially, BMP signaling is inhibited, resulting in induction of the pluripotency and EC factor SOX2, which promotes reprogramming of TCam-2 by induction of additional pluripotency and reprogramming factors [7]. Deletion of SOX2 interferes with this reprogramming, prolonging the seminoma fate of TCam-2 to six weeks [9]. In addition, in small subpopulations differentiation into non-seminomatous lineages has been observed. This in vivo differentiation was reminiscent of the in vitro differentiation observed by us, where TCam-2 were supplemented with media conditioned by murine fibroblasts and FGF4 (Figure 5A) [17]. In both cases, upregulation of germ layer marker genes (*AFP*, *PAX6*, *HAND1*) and marker genes indicative for extra-embryonic lineages (*EOMES*) was detected [9,17]. During the in vivo and in vitro differentiation, markers and morphology typical to an EC were not detected (Figure 5A) [9,17].

Since many upregulated differentiation factors interact with FOXA2, we hypothesized that FOXA2 might be instrumental to this differentiation [9,13,14,15]. By generating *SOX2*- and *FOXA2*-double deficient TCam-2 cells and xenografting of these cells into the somatic microenvironment of the murine flank, we demonstrated that FOXA2 is the factor essential for the induction of differentiation during in vivo growth of TCam-2. TCam-2-Δ*SOX2*/*FOXA2* showed no signs of reprogramming to an EC or differentiation into a mixed non-seminoma during the time observed (twelve weeks). The xenotransplanted cells maintained a seminoma-like gene expression profile and morphology. Our results establish FOXA2 (in addition to SOX2) as an essential factor driving reprogramming and differentiation of seminoma-like TCam-2. Both factors need to remain repressed to maintain a seminoma-like cell fate.

Expression of *FOXA2* is absent in the GCT cell lines TCam-2, 2102EP (EC), NCCIT (EC), and JAR (choriocarcinoma) as well as in the Sertoli cell line FS1 and adult fibroblasts (MPAF) (Figure S5A). Additionally, *FOXA2* expression is not detectable in seminomas, ECs, teratomas, mixed GCTs and normal testis tissues (NTT) (Figure S5B). Thus, we postulate that FOXA2 is only upregulated during differentiation of seminomas into non-seminomatous lineages and downregulated once adaptation to the newly acquired cell fate is completed. In light of our data, we also postulate that FOXA2 has no role in differentiation of ECs into teratoma, yolk-sac tumors and choriocarcinomas, since FOXA2 and FOXA2-associated differentiation factors are not upregulated during reprogramming of TCam-2 into an EC-like fate, in TCam-2-Δ*SOX2*, in 2102EP cells in vivo or in non-seminomatous tissues (Figure 4; Figure S4A,B) [7,9]. The fact that *FOXA2* is neither detected in 2102EP cells in vitro/in vivo nor in reprogrammed or in vitro cultured TCam-2 cells, but strongly upregulated in TCam-2-Δ*SOX2* in vivo [7,9] implies that induction of *FOXA2* is independent of *SOX2* expression. In contrast, a link between SOX17 and FOXA2 expression seems plausible. In mice, FoxA2 has been described as a direct transcriptional target of Sox17 [18]. In murine and human embryonic stem cells, Sox17/SOX17 is a known regulator of endodermal differentiation, but in humans SOX17 is also a master regulator of the primordial germ cell fate [6,19,20]. Furthermore, *SOX17* is highly expressed in seminomas, where it is thought to support pluripotency by functionally replacing *SOX2* [21]. During growth of TCam-2-Δ*SOX2* cells in vivo, in a subpopulation the role of SOX17 might switch from controlling seminomaness/pluripotency to a differentiation-inducing function (Figure 5B). As a consequence, FOXA2 is induced, which in turn drives differentiation into non-seminomatous lineage (Figure 5B). During further differentiation, *FOXA2*, *SOX17*, pluripotency and seminoma markers are downregulated (Figure 5B). This is in line with absent expression of *FOXA2* in non-seminomas (Figure S5A,B).

The trigger that initiates the switch in the role of SOX17 in the subpopulation remains to be identified. It might be a result of a contact to a different cell type in the microenvironment, providing different mitogens or growth factors. In summary, we provide evidence that FOXA2 induces the direct differentiation of seminoma-like TCam-2 cells into non-seminomatous cells in vivo.

## 4. Material and Methods

### 4.1. Ethics Statement

All animal experiments were performed according to the German law of animal protection and in agreement with the approval of the local institutional animal care committees (Landesamt für Natur, Umwelt und Verbraucherschutz, North Rhine-Westphalia (approval ID: AZ-84-02.04.2013-A430)).

### 4.2. Cell Culture

TCam-2 and 2102EP cells were cultivated as described previously [3,17].

### 4.3. Generation of FOXA2-Deficient TCam-2 Cells

TCam-2 cells homozygous deficient for *FOXA2* were generated as published [9]. Deletions within the coding sequence of *FOXA2* in each clone were detected by PCR (Figure S1A). See Table 1 for guideRNA and genotyping primer sequences.

### 4.4. DNA, RNA and Protein Isolation

Total RNA and proteins were isolated as described previously [8]. Briefly, RNA was isolated by the RNAeasy mini kit (Qiagen, Hilden, Germany) and proteins by RIPA buffer.

### 4.5. Western Blot

Western blots were performed as described previously [8]. Beta-ACTIN was used as housekeeper and loading control. See Table 2 for antibody details. Uncropped western blots are given in Figure S5C.

### 4.6. Hematoxylin and Eosin Staining

Four μm thick tumor tissue sections were deparaffinized in xylol for 2 × 10 min and then rehydrated decreasing ethanol concentrations (100% 5 min, 96% 3 min, 80% 3 min, 70%, 3 min). Afterwards slides were rinsed in tap water and stained in Hematoxylin (Merck, Darmstadt, Germany) for 3 min. Slides were again rinsed in tap water and then stained in Eosin (Merck) for 1 min. Samples were then incubated in increasing ethanol concentrations (70% 3 min, 80% 3 min, 96% 3 min, 100% 5 min) for dehydration. Slides were then incubated for 10 min in xylol and embedded with coverslips and Entellan (Merck).

### 4.7. Immunohistochemistry

Immunohistochemistry was performed as published [8]. Briefly, tumor tissues were dissected, fixed and processed in paraffin wax. Tissue sections on glass slides were pre-treated in the Lab Vision PT Modul (Thermo Scientific, Munich, Germany) and in PT Modul Buffer (pH 6) (TA-250-PM, Medac, Hamburg, Germany). Endogenous peroxidases were blocked by incubation in peroxidase blocking buffer (TA-125-HP, Medac) for 10 min. Primary antibodies were incubated for 30 min at room temperature. Signal detection was performed semiautomatically in the Autostainer 480 S (Medac) using the Bright Vision + polymer detection system (Medac) and the following settings: Enhancer for 10 min, polymer for 20 min, DAB (415192F, Medac) for 8 min. Afterwards, nuclei were stained by hematoxylin for 3 min. See Table 2 for antibody details and dilution ratios.

### 4.8. Quantitative RT-PCR

Quantitative RT-PCR (qRT-PCR) was performed as published previously [8]. 500 ng of total RNA was used for first strand synthesis. *GAPDH* was used as housekeeping gene and for data normalization. In general, all samples were analyzed in technical triplicates and biological triplicates/quadruplicates (see individual figure legend for more detailed information). See Table 1 for primer sequences.

### 4.9. Measurement of Proliferation Rates

Cell proliferation was determined by seeding 1 × 10^4^ cells/well. After 1, 3, 5, 7, 9 and 11 days cells were harvested by trypsinizing the cells and counted using a Neubauer counting chamber (BRAND, Wertheim, Germany). Cell numbers were determined in biological triplicates.

### 4.10. Xenotransplantation

Xenotransplantations of TCam-2 cells into the flank of nude mice were performed as described previously [22]. Briefly, 1 × 10^7^ tumor cells were dissolved in 500 μL Matrigel (Corning via VWR, Langenfeld, Germany) and injected using a syringe. Only male CD-1 nude mice (Crl:CD1-*Foxn1^nu^*) (Charles River, Erkrath, Germany) of six weeks of age were used.

### 4.11. Illumina HT-12v4 and Affymetrix Expression Arrays

The Illumina and Affymetrix expression array analyses of GCT cell lines and tissues were performed previously and re-analyzed in context of this study [3,7,9,23,24,25]. The microarray data sets are available via GEO (ncbi.nlm.nih.gov/geo/) (GSE76709; GSE71239).

## 5. Conclusions

In conclusion, SOX2 and FOXA2 are key factors in the reprogramming of seminomatous TCam-2 cells to an EC-like cell fate and differentiation into non-seminomatous lineages (except EC), respectively. Our results further strengthen the idea that seminomas show a plasticity, which is influenced by the microenvironment and regulated by SOX2 and FOXA2. These findings should now be confirmed in vivo by xenografting seminoma tissues.

## Figures and Tables

**Table 1:** Oligonucleotides used in this study.

Gene	Forward Primer (5′-3′)	Reverse Primer (5′-3′)
AFP	GTGGTCAGTTTGCAGCATTC	GCAGAGGAGATGTGCTGGAT
ALB	TCAGCCATTTCACCATAGGTT	TGCTGATGAGTCAGCTGAAA
APOA1	CCCAGTTGTCAAGGAGCTTT	TGGATGTGCTCAAAGACAGC
APOA2	AGTTCCGTTCCAGCCTTCTT	GACCGTGACTGACTATGGCA
DNMT3B	CCAGCTCTTACCTTACCATC	CAGACATAGCCTGTCGCTTG
DPPA3	TCAACGTCTCGGAGGAGATT	CAACCTACATCCCAGGGTCT
EOMES	CACATTGTAGTGGGCAGTGG	CGCCACCAAACTGAGATGAT
FOXA2	TACGTGTTCATGCCGTTCAT	CGACTGGAGCAGCTACTATGC
GAL	CTGGTGAGGCCATTCTTGTC	AAGGAAAAACGAGGCTGGA
GAPDH	TGCCAAATATGATGACATCAAGA	GGAGTGGGTGTCGCTGTTG
GDF3	CAGGAGGAAGCTGGGAAAT	TGCTACGTAAAGGAGCTGGG
ID1	TCCAGCACGTCATCGACTAC	TCAGCGACACAAGATGCG
ID3	TCAGCTTAGCCAGGTGGAAATC	TGGCTCGGCCAGGACTAC
LEFTY1	TTGGGGACTATGGAGCTCAG	TCAAGTCCCTCGATGGCTAC
LIN28A	ACCCTTCCATGTGCAGCTTA	TGTAAGTGGTTCAACGTGCG
NANOG	ATGGAGGAGGGAAGAGGAGA	GATTTGTGGGCCTGAAGAAA
NODAL	ATGCCAGATCCTCTTGTTGG	AGACATCATCCGCAGCCTAC
OCT3/4	GGGAGATTGATAACTGGTGTGTT	GTGTATATCCCAGGGTGATCCTC
PRAME	CGTAGACTCCTCCTCTCCCACAT	TGGGCGATATACTGCTCTTCCT
PRDM1	GGGTGCAGCCTTTATGAGTC	CCTTGTTCATGCCCTGAGAT
PRDM14	TCCACACAGGGGGTGTACTT	GAGCCTTCAGGTCACAGAGC
SOX17	GGCGCAGCAGAATCCAGA	CCACGACTTGCCCAGCAT
TFAP2C	GGCCCAGCAACTGTGTAAAGA	GCAGTTCTGTATGTTCGTCTCCA
FOXA2 genotyping 1	CCAGGGAGAGAGAGGGAGT	CCTCGGGCTCTGCATAGTAG
FOXA2 genotyping 2	CTCGCTCTCCTTCAACGACT	TCTTCTCCCTTGCGTCTCTG
FOXA2 genotyping 3	TTAAACTGCCATGCACTCGG	GGGAGTACACCCCCTGGTAG
FOXA2 guideRNA1	AAGGGCACGAGCCGTCCGAC	
FOXA2 guideRNA2	GTAGTGCATCACCTGTTCGT	
FOXA2 guideRNA3	CATGAACATGTCGTCGTACG	

**Table 2:** Antibodies used in this study.

**Primary Antibodies**					
**Target**	**Company**	**Number**	**Species**	**IHC**	**Western Blot**
AFP	Dako	A0008	Rabbit	1:250	-
β-Actin	Merck	A5441	Mouse	-	1:25000
EOMES	Abcam	ab23345	Rabbit	1:200	-
FOXA2	R&D systems	AF2400	Goat	1:200	1:500
Ki67	Zytomed Systems	MSK018	Mouse	1:500	-
OCT3/4	Santa Cruz	C-10	Mouse	1:200	-
PRAME	Santa Cruz	H-10	Mouse	-	1:400
PRDM1	H.M. Jäck	-	Rabbit	1:200	-
SOX2	R&D systems	MAB2018	Mouse	1:200	1:200
SOX17	Abcam	ab84990	Mouse	1:400	-
SOX17	R&D systems	AF1924	Goat	-	1:1000
TFAP2C	Santa Cruz	6E4/4	Mouse	1:200	-
**Secondary Antibodies**					
**Target**	**Company**	**Number**	**Species**	**IHC**	**Western Blot**
Rabbit Anti-Goat HRP	Dako	P0160	Rabbit	-	1:2000
Rabbit Anti-Mouse HRP	Dako	P0260	Rabbit	-	1:1000

## Supplementary Materials

The following are available online at https://www.mdpi.com/2072-6694/11/5/728/s1. Figure S1: Validation of a successful *FOXA2* gene editing, Figure S2: Measurement of proliferation rates of parental TCam-2, TCam-2-Δ*SOX2* and TCam-2-Δ*SOX2*/*FOXA2* cells over eleven days, Figure S3: A *SOX2*- and *SOX2*/*FOXA2*-deficiency does not influence the seminoma-like cell fate of TCam-2 cells in vitro, Figure S4: HE and IHC staining of SOX2, FOXA2, the pluripotency and seminoma markers OCT4, SOX17, TFAP2C and PRDM1 (green), the differentiation markers AFP and EOMES (red) and the proliferation marker Ki67 (blue) in TCam-2-ΔSOX2/FOXA2 tumor tissues twelve weeks after xenografting, Figure S5: Expression of *FOXA2* in GCT cell lines and tissues as well as supplemental western blot data.
