# Supplementary material for: TCam-2 Cells Deficient for SOX2 and FOXA2 Are Blocked in Differentiation and Maintain a Seminoma-Like Cell Fate In Vivo
Source: Cancers (Basel). 2019 May 25;11(5):728. doi: 10.3390/cancers11050728 (PMC6562827; doi:10.3390/cancers11050728)
Supplement: Supplementary file 1 [file cancers-11-00728-s001.pdf]

Supplementary Materials

TCam-2 Cells Deficient for SOX2 and FOXA2 Are Blocked in Differentiation and Maintain a Seminoma-Like Cell Fate In Vivo

Daniel Nettersheim, Saskia Vadder, Sina Jostes, Alena Heimsoeth and Hubert Schorle

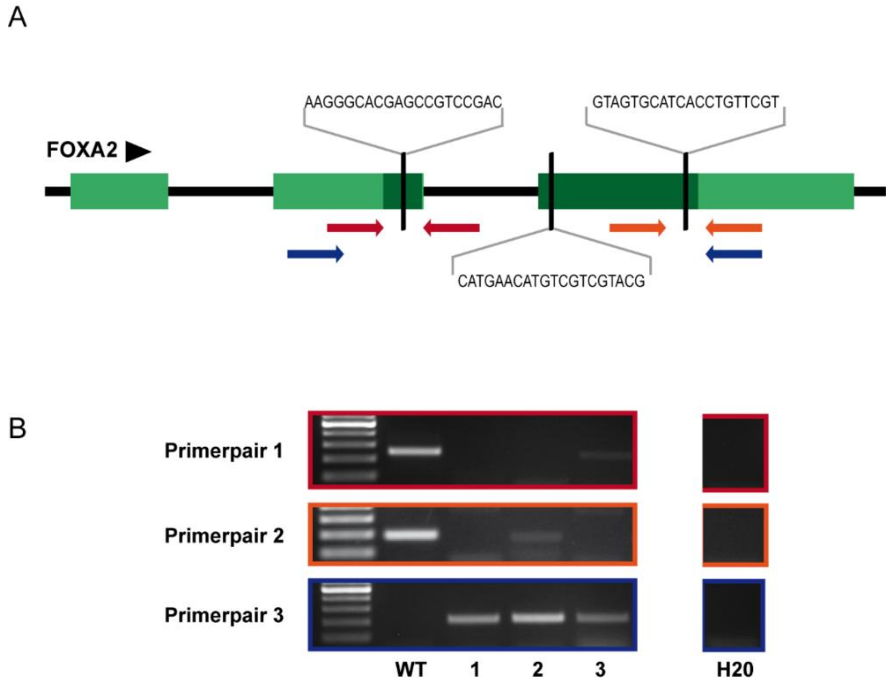

Figure S1. Validation of a successful *FOXA2* gene editing. (**A**) Schematic of the CRISPR/Cas9 strategy to knock out *FOXA2* in TCam-2 cells. Location of guideRNA1–3 and primer pairs for genotyping PCR are depicted. (**B**) Genotyping PCR using all three primer pairs depicted in (**A**). Three clones, already deficient for *SOX2* [9], show also deficiency for *FOXA2*.

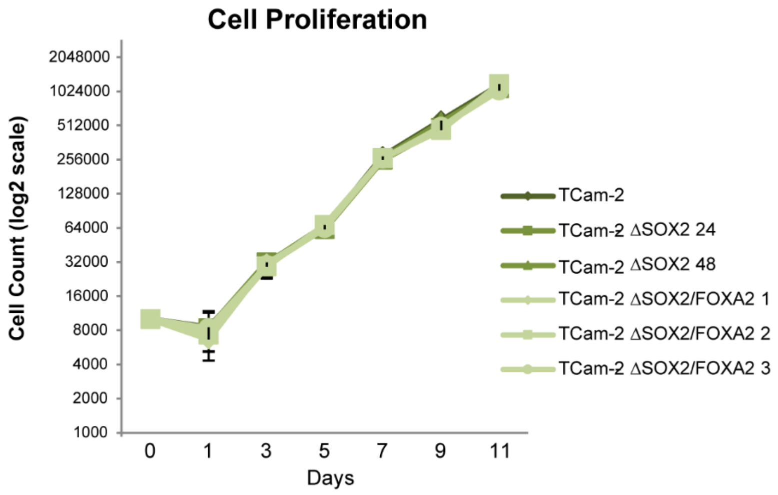

Figure S2. Measurement of proliferation rates of parental TCam-2, TCam-2-Δ*SOX2* and TCam-2-Δ*SOX2*/*FOXA2* cells over eleven days.

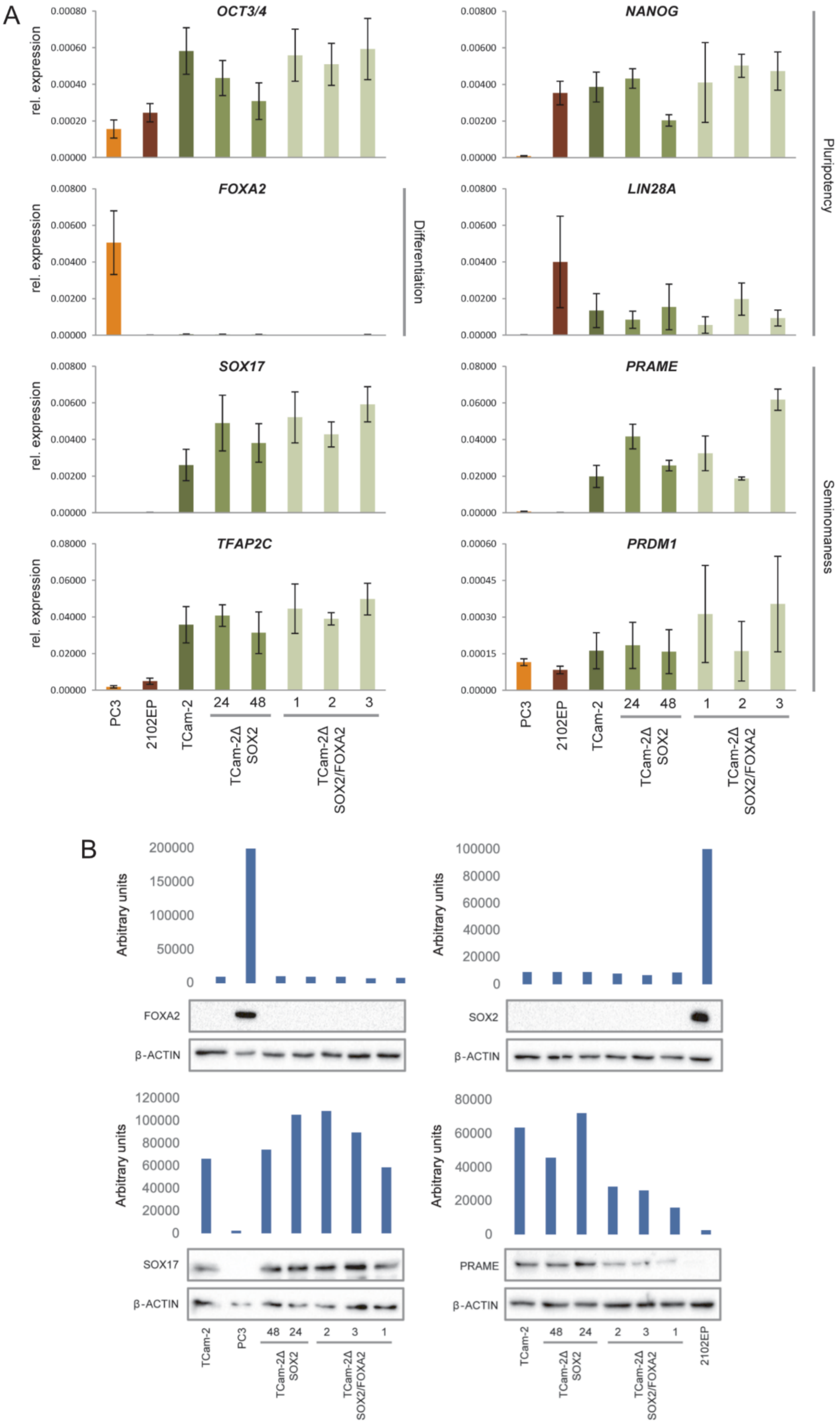

Figure S3. A *SOX2*- and *SOX2/FOXA2*-deficiency does not influence the seminoma-like cell fate of TCam-2 cells in vitro. (**A**,**B**) qRT-PCR (**A**) and western blot (**B**) analysis of indicated marker genes in

parental TCam-2, TCam-2-Δ*SOX2* and TCam-2-Δ*SOX2*/*FOXA2* cells. 2102EP and PC3 cells served as additional controls. Densitometrical analysis of western blot data is given above bands (**B**).

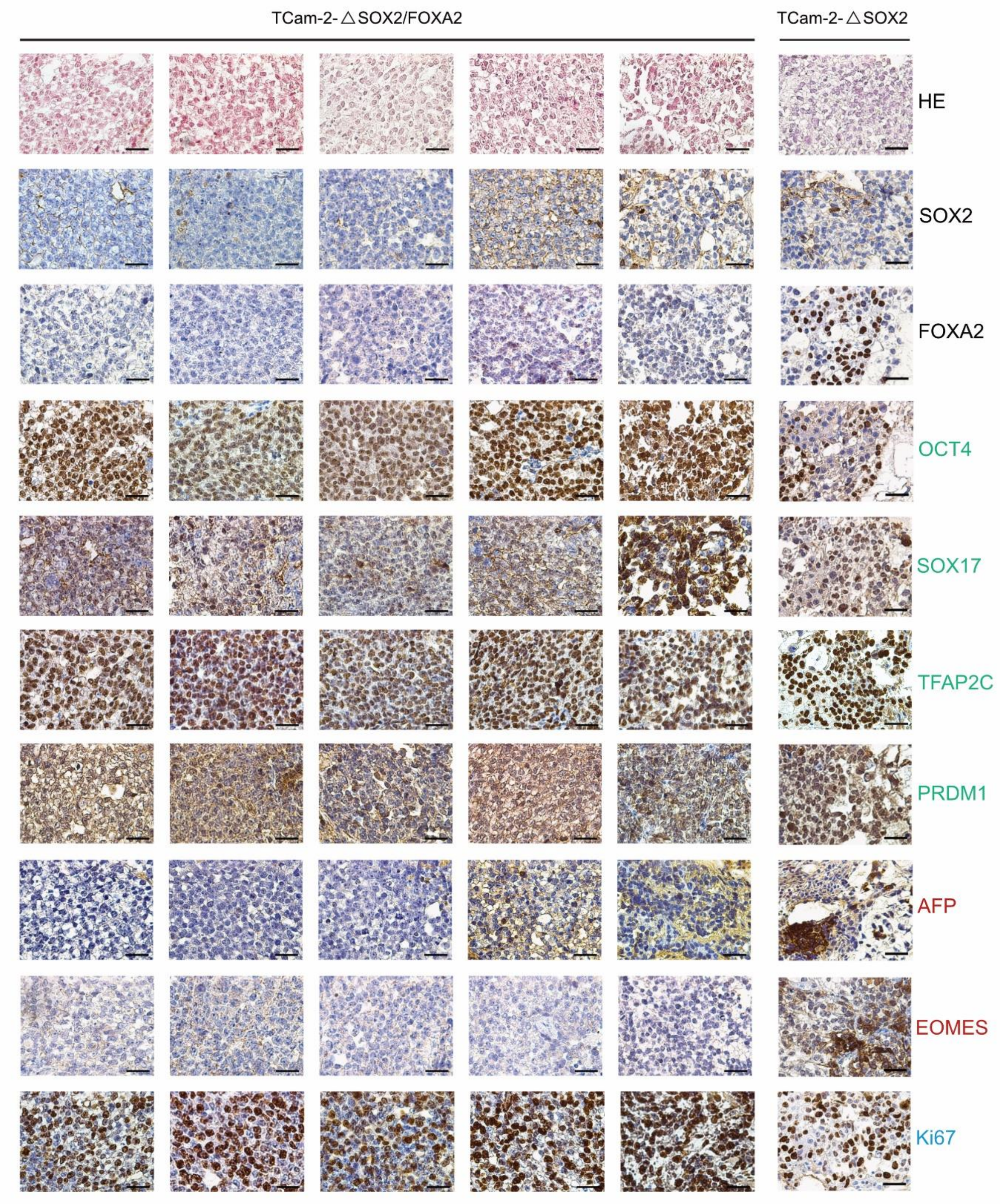

Figure S4. HE and IHC staining of SOX2, FOXA2, the pluripotency and seminoma markers OCT4, SOX17, TFAP2C and PRDM1 (green), the differentiation markers AFP and EOMES (red) and the proliferation marker Ki67 (blue) in TCam-2-ΔSOX2/FOXA2 tumor tissues twelve weeks after xenografting. Tumor tissue from TCam-2-ΔSOX2 cells served as control. Scale bars: 200 μm.

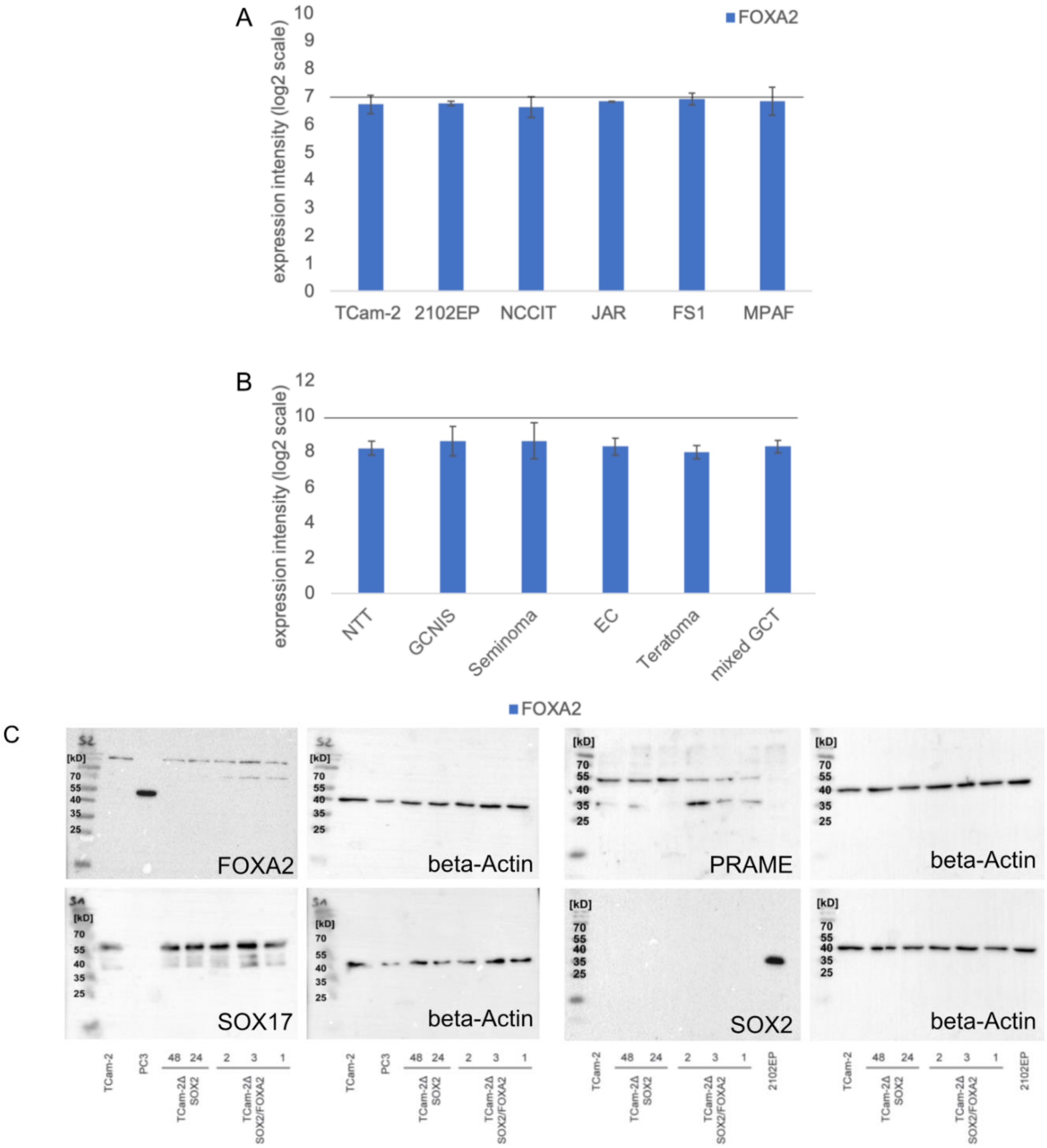

Figure S5. Expression of *FOXA2* in GCT cell lines and tissues as well as supplemental western blot data. (**A**,**B**) Microarray expression analysis of *FOXA2* expression in GCT cell lines (**A**) and tissues (**B**). Black lines indicate the minimum level of expression intensity that can be considered as 'expressed'. Differences in these thresholds is because of use of different microarray platforms: (**A**) Illumina, (**B**) Affymetrix. (**C**) Uncropped western blots of Figure S3 B including molecular weight markers.

© 2019 by the authors. Licensee MDPI, Basel, Switzerland. This article is an open access article distributed under the terms and conditions of the Creative Commons Attribution (CC BY) license (http://creativecommons.org/licenses/by/4.0/).
